# A concept mapping study evaluating the UK's first NHS generic fatigue clinic

**DOI:** 10.1111/hex.12405

**Published:** 2015-09-01

**Authors:** Katie L. Hackett, Rebecca L. Lambson, Victoria Strassheim, Zoe Gotts, Vincent Deary, Julia L Newton

**Affiliations:** ^1^ CRESTA Fatigue ClinicNewcastle upon Tyne Hospitals NHS Foundation TrustNewcastle upon TyneUK; ^2^ Musculoskeletal Research GroupInstitute of Cellular MedicineNewcastle UniversityNewcastle upon TyneUK; ^3^ Faculty of Medical SciencesNewcastle UniversityNewcastle upon TyneUK; ^4^ Institute of Health and SocietyNewcastle UniversityNewcastle upon TyneUK; ^5^ Department of PsychologyNorthumbria UniversityNewcastle upon TyneUK

**Keywords:** Fatigue, Chronic diseases, Patient expectations

## Abstract

**Importance:**

Fatigue is a significant and debilitating symptom affecting 25% of the population. It occurs in those with a range of chronic diseases, can be idiopathic and in 0.2–0.4% of the UK population occurs in combination with other symptoms that together constitute chronic fatigue syndrome (CFS). Until recently, NHS clinical services only focussed upon CFS and excluded the majority of fatigued patients who did not meet the CFS diagnostic criteria. The CRESTA Fatigue interdisciplinary clinic was established in 2013 in response to this unmet need.

**Objective:**

To identify the service needs of the heterogeneous group of patients accessing the CRESTA Fatigue Clinic, to prioritize these needs, to determine whether each is being met and to plan targeted service enhancements.

**Design:**

Using a group concept mapping approach, we objectively identified the shared understanding of service users accessing this novel clinic.

**Setting:**

NHS Clinics for Research & Service in Themed Assessment (CRESTA) Fatigue Clinic, Newcastle Upon Tyne, UK.

**Participants:**

Patients (*n* = 30) and referrers (*n* = 10) to the CRESTA Fatigue Clinic contributed towards a statement generation exercise to identify ways the clinic could support service users to improve their quality of life. Patients (*n* = 46) participated in the sorting and rating task where resulting statements were sorted into groups similar in meaning and rated for ‘importance’ and ‘current success’.

**Main outcome and measure:**

We mapped the needs of patients attending the CRESTA Fatigue Clinic and identified which high‐priority needs were being successfully met and which were not.

**Results:**

Multidimensional scaling and hierarchical cluster analysis depicted the following eight themed clusters from the data which related to various service‐user requirements: ‘clinic ethos’, ‘communication’, ‘support to self‐manage’, ‘peer support’, ‘allied health services’, ‘telemedicine’, ‘written information’ and ‘service operation’. Service improvement targets were identified within value bivariate plots of the statements.

**Conclusion and Relevance:**

Service development concepts were grouped into thematic clusters and prioritized for both importance and current success. The resulting concept maps depict where the CRESTA Fatigue Clinic successfully addresses issues which matter to patients and highlights areas for service enhancement. Unmet needs of patients have been identified in a rigorous service evaluation, and these are currently being addressed in collaboration with a service‐user group.

## Introduction

Chronic diseases will be the leading cause of disability by 2020, and despite medical advances leading to improved life expectancy, the symptoms experienced by those with chronic diseases will continue to become the most costly problem and greatest challenge facing health care.^1^ In the UK, 17.5 million adults are living with a chronic disease and 6 in 10 report some form of chronic disease a figure that continues to rise.^2^ Despite enhanced health care leading to improvements in chronic disease management, there is increasing realization that non‐disease‐specific symptoms persist in many people.

Fatigue is a debilitating symptom affecting all ages and can impact heavily upon quality of life. It can occur in association with a range of chronic diseases, can be unexplained (chronic fatigue or idiopathic fatigue) or occur with a constellation of symptoms that form chronic fatigue syndrome (CFS). Studies confirm that 25% of primary care attendances are attributable to fatigue, with it being the main reason in 6.5% of consultations.^3^ A British survey of those attending general practice found over 10% of adults had experienced substantial fatigue for over a month.^4^ In the United States, workers with fatigue cost their employers 136.4 billion dollars annually, an excess of 101 billion dollars compared with workers without fatigue. When fatigue occurs in association with other conditions, the condition‐specific lost productive time increases threefold.^5^

A recent audit of the regional CFS service in Newcastle upon Tyne, UK, identified 40% of referred patients with suspected CFS were not eligible for non‐pharmacological therapy as they did not fit the diagnostic criteria,^6^ a prerequisite for multidisciplinary therapy.^7^ A gap in service was evident as many people with long‐term physical conditions live with debilitating fatigue on a daily basis and are excluded from existing therapy services on the grounds of their diagnosis. Furthermore, a subset of CFS patients experience dysautonomia symptoms including postural orthostatic tachycardia syndrome and postural hypotension, which requires specialist multidisciplinary management.^8^, ^9^, ^10^ Patients with complex needs require services to be configured to prioritize their needs rather than those of health‐care systems.^11^ Subsequently, the Clinics for Research & Service in Themed Assessment (CRESTA) Fatigue Clinic was established to address this service gap with the implicit aim of meeting the needs of patients with chronic fatigue, irrespective of their physical health diagnosis. It is based on a Dutch model.^12^ The CRESTA Fatigue Clinic is an interdisciplinary clinic with occupational therapy, physiotherapy, sleep therapy, health psychology, nursing and medicine. To our knowledge, it is the first of its kind in the UK NHS. Consequently, demand for the service is high, with patients travelling long distances to be seen. Although patients accessing the service are offered therapy if appropriate, it remains unclear whether the service offered is meeting their expectations or requirements, without involving them fully in a comprehensive planning and evaluation project. This is vital to determine both the importance of individual expectations and the success of whether these expectations are being met.

The objectives of the service evaluation were to identify the service needs of the heterogeneous group of patients accessing the clinic, to prioritize these needs, to determine whether each need is being met and then to plan targeted service‐user‐informed service enhancements.

## Methods

Group concept mapping methodology (GCM), developed by Trochim,^13^ is a mixed‐methods participatory approach which uses a combination of group processes (brainstorming, sorting, rating and interpretation) and a sequence of multivariate statistical analysis steps (multidimensional scaling and hierarchical cluster analysis) that result in concept maps. Concept maps are visual representations of how participants conceptualize the relationship between ideas which they have generated on a particular topic. Priority values are added by participants to qualitative statements gathered during the brainstorming phase, and these can be interpreted in pattern matches and value plots and used in planning or evaluation studies.^14^

Group concept mapping typically involves five distinct phases:

1. Ideas generation/brainstorming

Participants who have a common area of interest are asked to provide their opinion in response to a focus prompt. Typically, a focus prompt is an incomplete sentence which stakeholders complete as many times as they like. This results in a list of ideas/statements.

Statement reduction

Duplicate statements are removed, and the refined statement list is checked for syntax and readability by the research team.

Sorting activity

Each statement is numbered and printed onto an individual card. Participants are asked to sort all the statements into piles of similar meaning statements and to give each pile a name. Participants record on a sheet the name of each pile they have created and statement numbers of the cards they contain.

Rating activity

Participants are given a list of the numbered statements and are asked to give each statement a value judgement (such as ‘importance’) on a 1–5 Likert scale.

Data analysis

The sorted data from each participant are entered into a software package specifically designed for GCM projects (Concept Systems Global Max™). Through a mathematical process of multidimensional scaling and hierarchical cluster analysis, concept maps are generated. Statements are represented by numbered points on the map. Statements that have been sorted together (often by participants) will be close together on the map and will be similar in meaning. Statements that are infrequently sorted together by participants will be located further from each other on the map and will be less similar in meaning. The cluster analysis results in clusters on the map which contain similar meaning statements and represent how the group see the ideas which they have generated. The software suggests names for the clusters based on the names participants gave to their piles in the sorting exercise.

The rating data are then analysed and used to produce ‘pattern matches’ and ‘go‐zones’. Pattern matches represent the value judgement for the rating scales for each of the generated cluster themes. Go‐zones are bivariate value plots which can be used to prioritize the statements. Both pattern matches and go‐zones can be used to evaluate and prioritize the clusters/themes and individual statements within them.

A strength of GCM over other approaches is that it is considered to be an equitable process, giving an equal voice to all participants, does not direct them to form a consensus and can be conducted with relatively large numbers of people representing different stakeholder groups. GCM offers an alternative approach to traditional open‐ended service evaluation questionnaires or face‐to‐face interviews. These require word‐analysis approaches and forced category classifications which inherently will have some level of researcher bias.^15^ In GCM, the statement data are classified into themes or clusters using a mathematical process which incorporates each participants’ sorting data equally and can be used to observe the combined value judgements of all participants. GCM uses a structured mixed‐methods approach to capture ideas from an identified group.^16^

GCM has been used in a variety of health‐care settings to plan, evaluate and make improvements to existing policy, interventions and services. This includes public health^17^, ^18^, ^19^, ^20^ rheumatology,^21^, ^22^ mental health,^23^, ^24^ cancer care^25^ and rehabilitation.^26^

This project was reviewed by the Research and Governance Department and was considered to be a service evaluation, meaning no research ethics committee permissions were required. The evaluation was registered as a service audit with the Newcastle upon Tyne Hospitals NHS Foundation Trust.

### The participants

Participants were identified in two distinct parts of the GCM process: during the ideas‐generation phase and again during the sorting and rating activities. First, all physicians (*n* = 55) who had referred patients to the CRESTA Fatigue Clinic received the brainstorming exercise by post. No identifiable patient information was provided. This was completed by 10 general practitioners and hospital consultants. Consecutive patients attending the CRESTA Fatigue Clinic took part in the anonymous brainstorming activity during their usual clinic attendance. The activity was made available in 12 consecutive clinics for patients to complete and place in a box in the waiting area. This was completed by 30 participants.

Secondly, the sorting and rating activities were posted out to 147 patients who had attended at least one appointment at the CRESTA Fatigue Clinic over its first year. Forty‐six took part in the sorting and/or rating tasks giving a return rate of 32%.

To develop a formal action plan, the final results were reviewed in consultation with a trained group of Health Champions who were ex‐patients specifically recruited from the CRESTA Fatigue Clinic in a project undertaken in collaboration with NHS England North and Altogether Better.^27^ Our Health Champions are a team of ex‐patients who have been brought together by a team from NHS England North, trained and empowered to feedback regarding their own and others’ experiences of attending the clinic and to develop their own strategies to enhance the clinic experience.

### Data collection

#### Generation of ideas (brainstorming)

In the first stage of the study, we asked referrers and clinic patients to complete the following sentence as many times as they could on a piece of paper:

A specific way the CRESTA Fatigue Clinic could support me to improve my quality of life is…

The resulting statement set was reduced to a shorter list of unique ideas^14^ with each statement coded with a keyword. Groups of statements containing the same keyword were considered in turn by the authors. Duplicate ideas were removed, statements that described the same or overlapping ideas were combined, and the resulting statement list was edited for syntax, and to ensure it would be easily understood. For example, *‘*Making it possible to discuss symptoms as they arise if GP can't find anything wrong’ was rephrased to ‘an opportunity to discuss my symptoms’. This process resulted in a synthesized statement list of 78 unique ideas (Table S1).

#### Structuring the ideas: rating and sorting

The refined statement set was mailed to 147 patients. They were asked to rate each statement on two dimensions – (i) *IMPORTANCE* and (ii) *CURRENT SUCCESS* on 1–5 rating scales. The instructions for the importance rating were ‘rate each statement below on how IMPORTANT you think it is by putting a circle around a number’. The number 1 on the rating scale represented ‘relatively unimportant’ and 5 ‘extremely important’. The current success rating instructions were ‘rate each item according to how successful the CRESTA Fatigue Clinic is at addressing that need currently’, and 1 on the scale represented ‘Need not being met at all’ and 5 ‘Need is successfully met’.

These patients were also given the option of completing a sorting task. The recommended number required for the sorting activity is 25.^16^ Because this task is time‐consuming, we expressed this activity was optional. Each statement was given a number and printed on a separate card. Participants were asked to sort the cards into groups that they felt were similar in meaning. Participants were then instructed to give each group of statements a name and to document them on a recording sheet with the corresponding statement numbers.

#### Analysing the data

The analyses were performed using Concept Systems Global Max™ software, specifically designed for GCM projects. Firstly, multidimensional scaling techniques were performed on the card sorting data, which had been arranged into a similarity matrix to position each statement in relation to each other as a point on an *x*–*y* axis. This generated a point map with each point representing a statement. The stress value, a statistic that reflects stability within the overall map, was generated. Instability in the point locations within the map occurs as the location of the points is calculated in multiple dimensions and is being confined to two‐dimensional space for easy visual representation. Ideal stress values for concept mapping analyses are below 0.36.^13^ Secondly, hierarchical cluster analysis was performed using Ward's algorithm which created clusters of statements. The clusters were examined by the authors, and maps containing as many as 16 clusters and as few as 4 were examined in an initial interpretation session in a process described by Kane and Trochim.^14^ In this process, the software was utilized to combine clusters one at a time by the authors. The statements within each cluster were examined to ensure they conveyed an overall theme. This is a qualitative decision made when it no longer makes sense to proceed to the next iteration as the contents of the cluster would be conceptually too broad. A provisional cluster solution was decided upon by the authors through discussion and agreed upon by our group of Health Champions.

Next, the rating data were considered. Importance and current success ratings were examined both at cluster level in a pattern match and at statement level in a go‐zone. The pattern match evaluated both importance and success of the themed clusters. The go‐zone allows the statements to be compared in a bivariate plot with mean rating scores for both importance and current success as cut‐offs to highlight zones. Statements falling above the mean cut‐off score received above‐average scores by participants, and those that fall below the mean cut‐off score received below‐average scores. The zones of particular interest were high importance and high current success (priority needs that are being successfully met) and high priority and lower current success (priority targets for service enhancement). The individual priority targets for service enhancements have been addressed by teams of service‐user Health Champions and clinic staff.

## Results

Ten referrers and 30 individuals attending the CRESTA Fatigue Clinic completed the brainstorming exercise, generating 154 statements that were subsequently distilled to a final statement set of 78. As the brainstorming was anonymous, we do not have participants’ demographic information. Forty‐six participants took part in stage two (sorting and rating). The mean age was 62.25 (SD 10.83). Of the 46 participants, 31 completed the sorting task, 46 completed the importance rating, and 40 completed the current success rating. Six participants opted not to complete the current success rating as they had only attended the clinic once and did not feel able to comment.

### Concept maps

**Figure 1:**
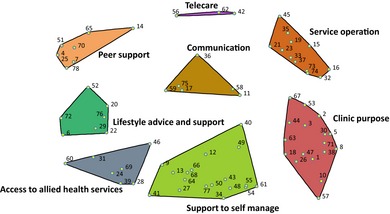
An eight‐cluster map showing the distribution of the individual statements as numbered points.

Multidimensional scaling resulted in a point map with a stress value of 0.24. An eight‐cluster solution was agreed upon the following: (i) *clinic purpose*; (ii) *support to self‐manage*; (iii) *peer support*; (iv) *lifestyle advice and support*; (v) *access to allied health services;* (vi) *communication*; (vii) *telecare*; and (viii) *service operation*. The smallest cluster (Telecare) contained three statements and the largest (Support to self‐manage) 18 statements. The point cluster map can be seen in Fig. 1. Here, each statement is represented by a numbered point on the map (individual statements can be viewed in Table S1). The points are grouped into the named clusters:

*Clinic Purpose*: The statements within this cluster represent the key purpose for the clinic. Typical statements within this cluster include ‘#8 – Take my fatigue seriously’, ‘#1 – Improve my health’, ‘#71 – Look at my symptoms as a whole’ and ‘#18 – Deliver a personalised treatment programme’ and ‘#63 – Being offered a physical assessment’.
*Support to self‐manage*: This is the largest cluster containing 18 statements. These include accessing support from a multidisciplinary team of health professionals who have expertise in fatigue (e.g. ‘#40 – Provide access to a dedicated team of professionals (medical, cognitive behavioural therapy, occupational therapy and physiotherapy) with expertise and an interest in fatigue’). Other statements within this cluster relate to support to manage specific symptoms such as dizziness (#55), pain (#61) and anxiety (#77) as well as support to make behavioural changes to increase activity/exercise levels (#68) and on balancing daily activities and rest (#66).
*Peer support*: Statements within this cluster relate to support patients could potentially give and receive from each other and include the following statements: ‘#4 – Make it possible for me to be in touch with others who have fatigue’ and ‘#25 – Meet other people with similar problems and learn how they manage’.
*Lifestyle advice and support*: Statements such as ‘#6 – Support with managing work and/or education’ and ‘#72 – Advice about financial support’ were representative of statements within this cluster.
*Access to allied health services*: This cluster included statements regarding accessing non‐medical health‐care professionals including occupational therapy (#46), physiotherapy (#24) and psychological support (#31 and #69) and access to specialist exercise programmes (#39, #28 and #60).
*Communication*: This cluster is at the centre of the concept map, indicating its central theme. The central location indicates that the clusters’ statements will have regularly been sorted with other statements within all surrounding clusters, despite it being a unique cluster. Statements include ‘#11 – Provide a culture of understanding which leaves patients understood and less isolated’ and more concrete forms of communication such as providing written information about fatigue and interventions (#58) and letters in support of adjustments within the workplace (#36) and benefits claims (#59).
*Telecare*: This is the smallest cluster and contains only three statements all relating to remote clinic access, including by telephone (#42), by Skype or similar (#62) and online drop in clinics (#56).
*Service operation*: This cluster contains statements about how the clinic is run including appointments (#74, #32 and #35), training medical students within the clinic about fatigue for future doctors (#19) and offering opportunities for clinic patients to participate in research projects (#21)

**Figure 2:**
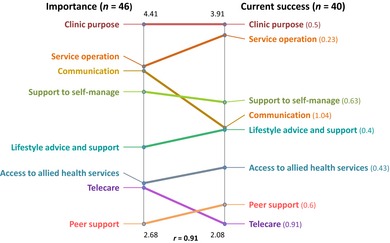
Pattern match demonstrating rating results at cluster level. Mean scores for statements falling within each cluster are demonstrated and clusters that were given comparatively higher scores are positioned nearer the top of the pattern match.

**Figure 3:**
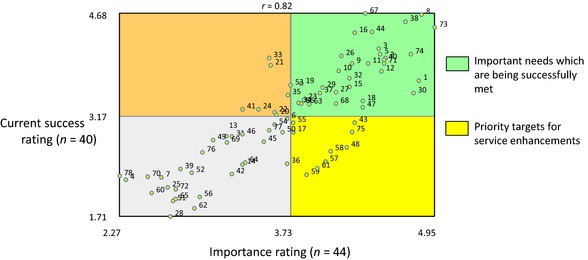
Go‐zone of all statements demonstrating rating results for each individual statement. Important needs that are being successfully met. Priority targets for service enhancements.

Comparisons between importance and current success ratings of the themed clusters can be seen in a ‘pattern match’ (Fig. 2). The correlation between ratings was high (*r* = 0.91). A ‘go‐zone’ bivariate plot was created which demonstrates the importance of each individual statement and whether it is being successfully met or not (Fig. 3). Important needs that are being met fall within the green quadrant of the go‐zone (Table 1). Important needs that are not being met falling within the yellow quadrant of the go‐zone are service enhancement targets (Table 2).

**Table 1:** Important statements/needs that were met successfully by the CRESTA Fatigue Clinic

Statement number	Statement	Mean current success rating (1–5)
67	Face to face contact	4.69
8	Take my fatigue seriously	4.68
38	Believing my symptoms	4.58
73	Keep the clinic going	4.50
44	An opportunity to discuss my symptoms	4.44
16	Copy of letters to GPs to be sent to patients	4.43
3	Provide up to date advice	4.21
74	Offer follow up appointments	4.13
26	Help me to understand my symptoms	4.08
5	Provide me with a diagnosis	4.08
2	Inform me of my test results	4.08
40	Provide access to a dedicated team of professionals (medical, cognitive behavioural therapy, occupational therapy and physiotherapy) with expertise and an interest in fatigue	4.00
11	Provide a culture of understanding which leaves patients understood and less isolated	3.97
71	Look at all my symptoms as a whole	3.97
9	Inform me how I could make beneficial changes to some of my behaviours	3.97
10	Give me hope	3.85
12	Support me to better manage my symptoms	3.84
32	Appointments to review my progress periodically	3.77
1	Improve my health	3.71
19	Have medical students in clinic so they can learn about fatigue	3.69
15	Co‐ordinate appointments with different members of the clinic team on the same day	3.68
29	Provide support, encouragement and reassurance	3.64
37	Clinic staff to liaise with other clinicians and agencies (when appropriate)	3.59
27	Support me to manage my daily activities	3.57
30	Investigate potential causes of my fatigue and offer treatments for them	3.51
18	Deliver a personalised treatment programme	3.49
23	Provide information to referring doctors about the clinic and the referral criteria	3.42
63	Being offered a physical assessment	3.38
34	Assist me in reaching my unique goals	3.35
66	Advice on balancing activities and rest	3.35
68	Advice on how to increase activity/exercise levels without reaching burn‐out	3.35
47	Help to distinguish which symptoms are due to which condition	3.34

Statements rated high for both importance and current success scores are demonstrated.

**Table 2:** Important statements/needs that were not successfully met and are targets for service enhancements

Statement number	Statement	Mean current success rating (1–5)
59	Provide letters giving information on my health and abilities for benefit claims	2.36
61	Help to manage pain	2.49
57	Cure my problem	2.51
48	Help me cope with brain fog	2.73
58	Provide written information about my condition, available treatments and other helpful services	2.76
17	Help me to explain my symptoms to others to help them to better understand	3.00
75	Keep me informed of new research findings	3.05
55	Help me overcome my dizziness	3.08
43	Provide new ways to help me cope with the fatigue	3.11

Statements rated high for importance and low for current success are demonstrated.

## Discussion

This novel service is successfully meeting the majority of important needs and has highlighted some specific targets for service enhancement. Only a small number of priority needs were not being successfully met. Most targets are being addressed by clinic staff in consultation with Health Champions, with the exception of ‘#57 – cure my problem’ deemed not realistically achievable.

This study has highlighted that the novel service being delivered in the Newcastle CRESTA addresses a considerable unmet need. A recent audit of our CFS NHS Clinical Service confirmed that 40% of those referred with a presumed diagnosis of CFS did not meet the diagnostic criteria for this condition but had fatigue related to other conditions.^6^ In addition, there is considerable recognition that the increase in chronic disease is likely to have a significant burden upon health‐care utilization in the coming decades. Simply managing the chronic disease does not necessarily impact upon the symptoms experienced by those affected. Developing strategies that address specifically the symptoms experienced by individuals (such as fatigue) are vitally important, if the true benefits of chronic disease management are to be realized. In addition, NHS CFS/ME clinical services and the NICE guidelines^28^ do not recommend that individuals with CFS who experience symptoms such as postural dizziness or loss of consciousness (known to occur in almost 90% of CFS patients^29^) have specific testing for dysautonomia.

This study created a large number of statements suggesting considerable expectations of individuals attending the service. Their expectations were diverse and heterogeneous, underlying the necessity for a multidisciplinary approach to symptom management. The themed clusters cut across a range of areas and highlight the complexity of symptom and chronic disease management. It is vital that as chronic disease prevalence increases, the UK NHS develops models of care that are able to meet patients’ needs and address these complex issues.

The study confirms that the clinic model (despite being early in its conception and implementation) is addressing a large number of areas for this patient group. The statements rated highest and the most successful suggest that fatigue patients value the outpatient attendance. Some statements suggest that experiences in other clinical services may have been suboptimal (e.g. #8, ‘Take my fatigue seriously’; #38, ‘Believing my symptoms’; #10, ‘Give me hope’; #53, ‘Time in appointments to express my feelings’; #11, ‘Provide a culture of understanding which leaves patients understood and less isolated’). This is disappointing. Recognizing that these symptoms can be extremely debilitating and impact dramatically upon quality of life highlights the need to incorporate symptom management into current systems.

The holistic, multidisciplinary approach that is central to the CRESTA Fatigue Clinic in Newcastle is valued by the patient group (#40, ‘Provide access to a dedicated team of professionals (medical, cognitive behavioural therapy, occupational therapy and physiotherapy) with expertise and an interest in fatigue’), and one of the high importance/priority statements is the need for continued intermittent follow‐up (#74, ‘Offer follow up appointments’).

The sense of being cast free from an outpatient clinic for those with chronic disease can be extremely isolating. In the clinic, we are now working with our Health Champions to develop strategies that will fulfil some of the needs of this patient group beyond the immediacy of the clinical service. It is arguable that much of what this patient group require should be met in primary care. Currently, this appears not to be the case and there is a real need to develop clinical services that bridge between primary and secondary care for patients with chronic disease who have on‐going debilitating symptoms.

Clearly, the current NHS model of ‘ologies’ and the immediacy of ‘new to review’ ratios do not fit cleanly with the model that is desired by this patient group. If we are to avoid the revolving door element of chronic disease patient management, it is essential that different models of care are evaluated and implemented in order to meet the needs of this increasing patient group.

There are some needs patients feel the clinic does not meet. We, alongside Health Champions, are addressing these. Specifically for statements #59 and #58, we have developed generic fatigue information for patients attending the clinic, something which has not previously been available; for #61 and #48, we are increasing the information available regarding pain and the symptom of brain fog to aid self‐management; and for #75, #55 and #43, we have a very active research programme in Newcastle with the Newcastle Fatigue Research Centre. The patients who are seen in the clinic directly link into any on‐going research studies, and the results of these studies are disseminated back to those attending the clinic. This is clearly something that this patient group value enormously.

It is important to acknowledge some potential limitations of this study. This study began when the clinic was in its infancy. Because all patients who had accessed the service were invited to take part, they may have attended the clinic before there was a full complement of therapy staff. This may have affected the results. Despite this, there are only a small number of priority needs that were not being successfully met. Although one is not realistically achievable at this moment in time (#57, Cure my problem), the other targets are currently being addressed by clinic staff in partnership with our service‐user Health Champions who are acting as advocates for the patients attending the clinic and developing a peer support system.

The average age of respondents in this service review was 62.25; this is considerably older than that in CFS/ME‐specific clinics (males: 41.4, females: 38.6).^30^ As a result, their service needs may be different to younger patients. As the UK population ages, the demand for such clinics is likely to increase.

Concept mapping is a relatively recently developed methodology. It has some advantages in that it is considered to be an equitable process, giving an equal voice to all participants and does not direct them to form a consensus as in other methods such as Delphi. Once the data has been collected and inputted into the software, the quantitative analyses can be conducted often using a large sample size, relatively quickly prior to being presented to stakeholders for discussion and interpretation. It offers an alternative, systematic approach to traditional open‐ended service evaluation questionnaires or face‐to‐face interviews. A disadvantage is that it requires specialist training and a software licence which can be costly, depending on the size of the project. It the future, concept mapping could be used to track service changes and to facilitate co‐design of new services.

The return rate for the questionnaires was 32%; consequently, we must accept that the needs discussed may not be fully representative of all CRESTA patients. However, the required number for statistical analysis was exceeded.^16^

Overall, the UK's first NHS generic interdisciplinary fatigue clinic is a success. It provides patients with the access to a multidisciplinary approach who would otherwise not receive this level of care. The service users value the clinic and are keen for it to be continued. They have highlighted some specific areas for service enhancements which will tailor the service further to the needs of the patients.

## Authors’ contributions

KH developed the concept and designed the study. It was conducted by KH and RL. All authors contributed to the analysis of the data. JN supervised the project, and the manuscript was written and commented on by KH, JN and RL.

## Source of funding

This study was funded through a College of Occupational Therapists Innovation Award. The funders had no involvement in the design, conduct, data collection, data management, analysis, interpretation of the data or approval of the manuscript.

## Conflict of interest

None declared.

## Supporting information


**Table S1.** Mean priority ratings of all participants.
