# Supplementary material for: A concept mapping study evaluating the UK's first NHS generic fatigue clinic
Source: Health Expect. 2015 Sep 1;19(5):1138–49. doi: 10.1111/hex.12405 (PMC5054859; doi:10.1111/hex.12405)
Supplement: Supplementary file 1 — Table S1. Mean priority ratings of all participants. [file HEX-19-1138-s001.docx]

**Table 1.** Mean priority ratings of all participants. Importance and current success were scored for each statement on a 1-5 scale with 5 being the maximum rating score

| **Statement number** |  | **Importance mean (n=46)** | **Current Success mean (n=40)** |
| --- | --- | --- | --- |
|  | **CLINIC PURPOSE CLUSTER** |  |  |
| 8 | Take my fatigue seriously | 4.85 | 4.68 |
| 1† | Improve my health | 4.83 | 3.71 |
| 30† | Investigate potential causes of my fatigue and offer treatments for them | 4.78 | 3.51 |
| 38† | Believing my symptoms | 4.72 | 4.58 |
| 2† | Inform me of my test results | 4.56 | 4.08 |
| 71† | Look at all my symptoms as a whole | 4.54 | 3.97 |
| 5† | Provide me with a diagnosis | 4.51 | 4.08 |
| 3† | Provide up to date advice | 4.50 | 4.21 |
| 44† | An opportunity to discuss my symptoms | 4.44 | 4.44 |
| 67† | Face to face contact | 4.39 | 4.69 |
| 18† | Deliver a personalised treatment programme | 4.37 | 3.49 |
| 47† | Help to distinguish which symptoms are due to which condition | 4.37 | 3.34 |
| 26† | Help me to understand my symptoms | 4.20 | 4.08 |
| 10† | Give me hope | 4.17 | 3.85 |
| 57‡ | Cure my problem | 4.07 | 2.51 |
| 63† | Being offered a physical assessment | 3.85 | 3.38 |
| 53 | Time in appointments to express my feelings | 3.76 | 3.68 |
|  | **Mean cluster ratings** | **4.41** | **3.90** |
|  |  |  |  |
|  | **SERVICE OPERATION CLUSTER** |  |  |
| 73† | Keep the clinic going | 4.96 | 4.50 |
| 74† | Offer follow up appointments | 4.76 | 4.13 |
| 16† | Copy of letters to GPs to be sent to patients | 4.30 | 4.43 |
| 32† | Appointments to review my progress periodically | 4.26 | 3.77 |
| 15† | Co-ordinate appointments with different members of the clinic team on the same day | 4.22 | 3.68 |
| 37† | Clinic staff to liaise with other clinicians and agencies (when appropriate) | 4.00 | 3.59 |
| 23† | Provide information to referring doctors about the clinic and the referral criteria | 3.89 | 3.42 |
| 19† | Have medical students in clinic so they can learn about fatigue | 3.85 | 3.69 |
| 35 | Offer a range of appointment times | 3.65 | 3.56 |
| 21 | Opportunities for me to be involved in research studies | 3.60 | 3.97 |
| 33 | Provide a leaflet which explains about the clinic prior to my first appointment | 3.54 | 4.03 |
| 45 | Provide a clinic close to where I live | 3.48 | 2.87 |
|  | **Mean cluster ratings** | **4.04** | **3.80** |
|  |  |  |  |
|  | **COMMUNICATION CLUSTER** |  |  |
| 11† | Provide a culture of understanding which leaves patients understood and less isolated | 4.41 | 3.97 |
| 75‡ | Keep me informed of new research findings | 4.28 | 3.05 |
| 58‡ | Provide written information about my condition, available treatments and other helpful services | 4.07 | 2.76 |
| 59‡ | Provide letters giving information on my health and abilities for benefit claims | 3.85 | 2.50 |
| 17‡ | Help me to explain my symptoms to others to help them to better understand | 3.74 | 3.00 |
| 36 | Provide letters giving information on my health, abilities and suggested reasonable adjustments for my employer/place of education | 3.67 | 2.36 |
|  | **Mean cluster ratings** | **4.00** | **2.94** |
|  |  |  |  |
|  | **SUPPORT TO SELF-MANAGE CLUSTER** |  |  |
| **Statement number** |  | **Importance mean (n=46)** | **Current Success mean (n=40)** |
| 40† | Provide access to a dedicated team of professionals (medical, cognitive behavioural therapy, occupational therapy and physiotherapy) with expertise and an interest in fatigue | 4.52 | 4.00 |
| 12† | Support me to better manage my symptoms | 4.52 | 3.84 |
| 43‡ | Provide new ways to help me cope with the fatigue | 4.28 | 3.11 |
| 9† | Inform me how I could make beneficial changes to some of my behaviours | 4.24 | 3.97 |
| 48‡ | Help me cope with brain fog | 4.20 | 2.73 |
| 68† | Advice on how to increase activity/exercise levels without reaching burn-out | 4.13 | 3.35 |
| 27† | Support me to manage my daily activities | 4.09 | 3.57 |
| 61‡ | Help to manage pain | 3.91 | 2.49 |
| 34† | Assist me in reaching my unique goals | 3.83 | 3.35 |
| 66† | Advice on balancing activities and rest | 3.78 | 3.35 |
| 55‡ | Help me overcome my dizziness | 3.72 | 3.08 |
| 54 | Help me improve my balance | 3.57 | 3.05 |
| 50 | Help with my sleeping | 3.57 | 2.94 |
| 77 | Help me manage anxiety and stress | 3.46 | 3.03 |
| 41 | Provide me with exercises | 3.35 | 3.32 |
| 64 | Advice about diet | 3.35 | 2.47 |
| 13 | Inform me of relaxation and meditation techniques | 3.24 | 2.86 |
| 49 | Guidance on how best to take my medication | 3.07 | 2.89 |
|  | **Mean cluster ratings** | **3.82** | **3.19** |
|  |  |  |  |
|  | **LIFESTYLE ADVICE AND SUPPORT CLUSTER** |  |  |
| 29† | Provide support, encouragement and reassurance |  |  |
| 20 | Being able to talk to someone about anything | 4.04 | 3.59 |
| 6 | Support with managing work and/or education | 3.67 | 3.14 |
| 22 | Support me to manage life events such as change in working patterns or temporary lifestyle changes | 3.58 | 3.20 |
| 76 | Support for my family | 3.57 | 3.24 |
| 52 | Help to reduce my isolation | 3.00 | 2.65 |
| 72 | Advice about financial support | 2.83 | 2.35 |
|  | **Mean cluster ratings** | 2.74 | 2.11 |
|  |  | **3.35** | **2.90** |
|  | **ACCESS TO ALLIED HEALTH SERVICES CLUSTER** |  |  |
| 24 | Access to physiotherapy | 3.50 | 3.27 |
| 46 | Access to occupational therapy | 3.24 | 2.95 |
| 31 | Offer one to one counselling | 3.20 | 2.89 |
| 69 | Access to psychological therapy | 3.09 | 2.79 |
| 39 | Deliver an endurance exercise training programme | 2.84 | 2.42 |
| 28 | Provide massage and hydrotherapy sessions | 2.72 | 1.75 |
| 60 | Start a class to promote exercise | 2.63 | 2.08 |
|  | **Mean cluster ratings** | **3.03** | **2.59** |
|  |  |  |  |
|  | **PEER SUPPORT CLUSTER** |  |  |
| 14 | Access to a telephone or email helpline for advice and support when required | 3.39 | 2.47 |
| 51 | A group session for patients to be able to meet up with each other every few months | 2.76 | 2.06 |
| 25 | Meet other people with similar problems and learn how they manage | 2.74 | 2.16 |
| 65 | Online forums and/or discussion board for CRESTA patients to share stories | 2.72 | 1.97 |
| **Statement number** |  | **Importance mean (n=46)** | **Current Success mean (n=40)** |
| 7 | Advice about outside support/support groups | 2.67 | 2.38 |
| 70 | Provide access to an expert patient | 2.53 | 2.28 |
| 4 | Make it possible for me to be in touch with others who have fatigue | 2.38 | 2.39 |
| 78 | Being able to talk to someone who is not a patient or a doctor | 2.24 | 2.32 |
|  | **Mean cluster ratings** | **2.68** | **2.25** |
|  |  |  |  |
|  | **TELECARE CLUSTER** |  |  |
| 42 | Offer telephone appointments | 3.17 | 2.35 |
| 56 | Provide an online drop in clinic | 2.93 | 2.03 |
| 62 | Have the option for skype (or similar) appointments | 2.87 | 1.86 |
|  | **Mean cluster ratings** | **2.99** | **2.06** |

† indicates important statements that are being successfully met.

‡ indicates important statements that are service improvement targets.
